# Von Bergmann’s Surgery

**Published:** 1904-11

**Authors:** 


VON BERGMANN'S SURGERY. A system of Practical Surgery. Drs. E. Von Bergmann, of Berlin, P. Von Cruns, of Tubingen and J. Von Mikulizc, of Breslau. Edited by William T. Bull, M.D., Professor of Surgery in the College of Physicians and Surgeons (Columbia University), New York. Complete work now ready, in five imperial octavo volumes, containing 4220 pages, 1976 engravings and 102 full-page plates in colors and monochrome. Sold by subscription only. Per volume, cloth, $6.00; leather, $7.00; half morocco, $8.50, net. Volume V just ready. 789 pages, 354 engravings, 23 plates. Lea Brothers & Co., Publishers, Philadelphia and New York.

This volume covers malformation, injuries and diseases of the pelvis, the anus and rectum, the urethra, the penis, abnormalities, injuries and diseases of the kidneys and ureter, the bladder and prostate, the scrotum, testicles, vas deferens and seminal vesicle, etc., and completes the work. Although dealing with the pelvis as a whole there is no gynecological part. As the introduction above would seem to show, as well as an examination of the text, the diseases and injuries dealt with are chiefly exclusive of the female generative organs, but include diseases, etc., of the urinary organs. The illustrations and text are of the same general excellence as in the other volumes.

